# A Scoping Review to Identify Interventions That Support Healthier Food Choices for Pupils in Specialist Schools

**DOI:** 10.3390/nu18071037

**Published:** 2026-03-25

**Authors:** Suzanne Spence, Louise Tanner, João P. A. Greca, Lindsay Pennington, Jayne V. Woodside, Morag J. Andrew

**Affiliations:** 1 Population Health Sciences Institute, Faculty of Medical Sciences, Newcastle University, Newcastle Upon Tyne NE2 4HH, UK; louise.tanner@newcastle.ac.uk (L.T.); joao.greca@newcastle.ac.uk (J.P.A.G.); lindsay.pennington@newcastle.ac.uk (L.P.); morag.andrew@newcastle.ac.uk (M.J.A.); 2 Human Nutrition & Exercise Research Centre, Faculty of Medical Sciences, Newcastle University, Newcastle Upon Tyne NE2 4HH, UK; 3 NIHR Innovation Observatory, The Catalyst, 3 Science Square, Faculty of Medical Sciences, Newcastle University, Newcastle Upon Tyne NE4 5TG, UK; 4 Centre for Public Health, School of Medicine, Dentistry Biomedical Sciences, Institute of Clinical Sciences A, Queen’s University Belfast, Grosvenor Road, Belfast BT12 6BJ, UK; j.woodside@qub.ac.uk

**Keywords:** child nutrition, dietary interventions, food choices, school food, special education

## Abstract

**Background/Objectives:** Children and young people (CYP) with a learning disability are at higher risk of living with overweight and obesity and may consume fewer fruits and vegetables compared to the general paediatric population. They are more likely to experience eating and drinking difficulties, restrictive eating, and mealtime behavioural challenges. The school environment is considered an ideal setting to improve CYP’s dietary intakes. The primary objective was to identify existing interventions to support healthier food choices for CYP attending specialist schools. Secondary objectives considered intervention development, fidelity and outcomes. **Methods:** A scoping review and narrative synthesis. Eligible studies were identified from bibliographic databases (e.g., Medline, Embase, PsychInfo) and grey literature (e.g., Clinicaltrials.gov, the Cochrane Library). A two-stage screening process was used. Intervention components were mapped according to the TIDieR-PHP and AACTT frameworks. **Results:** Seven studies, reported in ten records, were included. Interventions included modifications to the dining environment, sensory exploration, health promotion and social reinforcement. Interventions were implemented across the school day: lunchtime (*n* = 2), breaktime (*n* = 3) and other times (*n* = 2). Studies mainly focused on adolescents. There was some mixed evidence of increased consumption of fruits and vegetables, whole grains and water. Due to small sample sizes and heterogeneity, definitive conclusions are limited. A key finding is the lack of interventions to improve CYP’s food choices in specialist schools. **Conclusions:** This review highlights a crucial need for the development of multi-component interventions co-produced with stakeholders to promote healthy food choices and improve the dietary intakes of CYP attending specialist schools.

## 1. Introduction

Childhood obesity is a global public health issue. In 2022, 390 million children and adolescents aged 5–19 years were classified as overweight, including 160 million living with obesity [1]. In England, roughly 10% of 4–5-year-olds and 20% of 10–11-year-olds were living with obesity in 2023/24 [2]. Childhood obesity is linked to diets high in fat and energy, decreased intake of fruits and vegetables, and increased sugar consumption [3]. Evidence shows that eating behaviours established during childhood track into adulthood [4], highlighting the importance of establishing healthy eating behaviours early in life. Food choices are influenced by many factors, including individual, social and physical environments and policies [5].

Compared to the general paediatric population, CYP with a learning disability are at even higher risk of living with obesity [6] and may consume fewer FVs [7]. Some will also experience underweight and undernutrition due to health conditions and/or eating and drinking difficulties that impact the quality and quantity of their nutritional intake [8]. In addition, complex behavioural challenges and sensory preferences leading to restrictive eating patterns may impact dietary intakes [9].

The school environment is often considered as an ideal setting to improve the dietary intakes of CYP, as most attend school. School food contributes to 30% of daily intake [10], and pupils come from across the socio-economic spectrum [11]. Specialist schools provide education to CYP with special educational needs (SENs) whose needs cannot be met within a mainstream setting [12]. There are four broad areas of need: communication and interaction, cognition and learning, social emotional and mental health, and sensory and physical needs [12]. In 2024, over 1.6 million primary and secondary school pupils in England accessed SEN provision [13]; of these, roughly 10% attend a specialist school [14] and have at least one health condition [13].

In the UK, school food standards specify what food and drink can be provided [15]. In addition to school food standards, there is research exploring the implementation of school-based interventions to improve CYP’s dietary intakes. A systematic review by Micha et al., 2018 [11] considered the effectiveness of school food environment policy interventions on CYP’s dietary behaviours and highlighted that these can have positive impacts. They found school food standard policies increased fruit intakes and reduced some key nutrient intakes, such as saturated fat and sodium. However, less evidence was found for adiposity outcomes. Similarly, Metcalfe et al., 2020 [16] undertook a systematic review of school nudge-based interventions and the effect on CYP’s food behaviours. They found these interventions improved pupils’ food selection but were less consistent for food consumption. For both reviews, included studies were conducted in mainstream primary and secondary schools, not specialist schools. This scoping review aimed to identify interventions that support healthier school food choices in CYP attending specialist schools to inform future opportunities for intervention development.

## 2. Materials and Methods

The protocol for this scoping review was uploaded onto the Figshare repository (December 2024) [17] and conforms to the Preferred Reporting Items for Systematic Reviews and Meta-analyses Protocols (PRISMA-P) [18]. The scoping review adheres to the PRISMA Scoping Review (PRISMA-ScR) checklist [19] (Supplementary File S1).

### 2.1. Objectives and Research Questions

The objective of this scoping review was to identify evidence-based interventions to support healthier food choices for children and young people (CYP) attending specialist schools. The research questions were as follows:

1. What evidence-based interventions exist to support healthier food choices for CYP attending specialist schools?
2. How are these interventions developed and implemented within specialist schools, and what are their impacts on dietary choices, nutritional intake, and health outcomes for CYP?

### 2.2. Search Strategy

The literature search was developed in Medline (Supplementary File S2), with support from information specialists. A variety of search terms were utilised, for example, (food* or nutri* or diet or eat*), (Learning Disabilities/Neurodevelopmental Disorders), (intervention* or program* or promot*) and (Schools/Education, Special); the complete search strategies, including all term combinations, are detailed in Supplementary File S2. The search strategy was adapted for Embase, PsychInfo, Scopus, ERIC and CINAHL. Sources of grey literature included Cochrane and Campbell libraries, Google Scholar and Clinical trials.gov. The reference lists of eligible studies were searched to identify additional publications. The searches were conducted between the 5 and 19 November 2024.

### 2.3. Eligibility Criteria

The eligibility criteria were defined according to the Population, Concept and Context (PCC) framework:

- Population: CYP with SENs, age range 4–22 years, and mean participant age ≤ 19 years.
- Concept: The intervention, including any programme, policy, or practice to improve dietary choices or nutritional intake.
- Context: Specialist schools. In some education systems, for example, the USA [20], CYP with SENs are not educated in separate specialist schools but instead attend mainstream schools. Consequently, some studies of relevance to our review were conducted in mainstream settings. To maintain consistency with our eligibility criteria, we included mainstream-based studies only when the participants received the intervention in segregated or self-contained SEN classrooms, as this mirrors specialist provision within those systems and is standard practice in countries such as the USA [20]. Interventions implemented in alternative provisions [21] or in the home were also excluded.
- Outcomes of interest were measures of healthy eating, including FV consumption and nutrient intake, and any reported health outcomes. No time or geographical restrictions were applied. Studies published in languages other than English were excluded. Non-English publications were excluded due to time constraints and a lack of translation resources.

### 2.4. Selection of Evidence

Identified records were downloaded into EndNote for deduplication and screened in Rayyan [22]. Reviewers screened 10% of titles and abstracts independently in duplicate, and discrepancies were resolved through discussion. Any remaining differences of opinion were resolved by discussion with the wider team. The remaining records were screened by a single reviewer. The same process was followed for full-text screening. When a full text was unavailable, the full text manuscript was requested from the author.

### 2.5. Data Extraction and Data Synthesis

Data were extracted into Excel to include citation details, study characteristics, participant characteristics (intervention group and control group), details of the control condition, intervention description and components according to elements from the Action, Actor, Context, Target, Time (AACTT) [23] and Template for Intervention Description and Replication for Population Health and Policy Interventions (TIDieR-PHP) [24] frameworks, and results, conclusions and limitations. The study designs were classified using the Design Algorithm for Medical Literature on Intervention (DAMI) tool [25] to provide a standardised framework for classifying study designs for medical literature. The data extraction tool was piloted on a sample of included studies to ensure that it captured all the required information for the synthesis. One reviewer extracted data, and a second reviewer checked data for accuracy. A narrative synthesis was undertaken. Studies were grouped into categories based on intervention type (e.g., modification to the dining environment), providing a structured approach. Critical appraisal was not conducted because the primary aim of this scoping review was to identify existing interventions and, secondarily, to map their components. However, limitations of the included studies were considered.

## 3. Results

**Figure 1:**
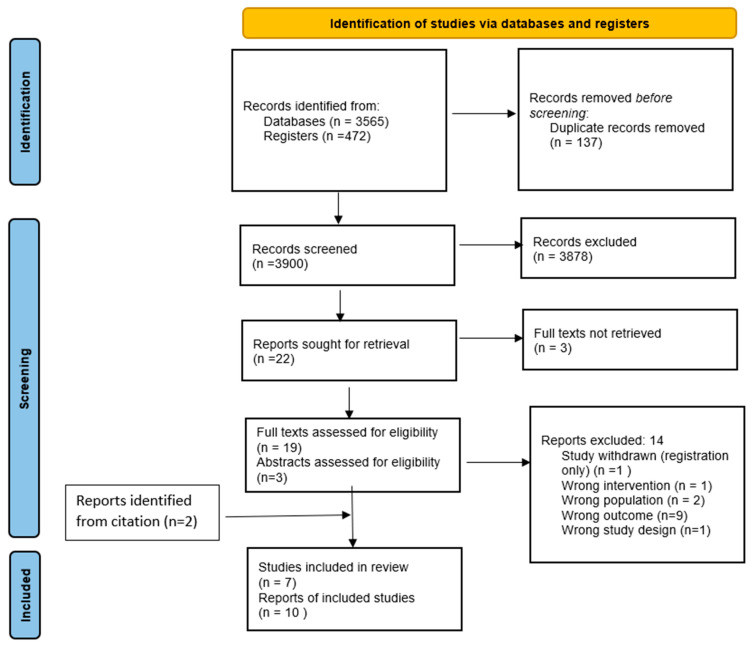
PRISMA flow diagram depicting the flow of included and excluded studies.

A total of 4037 records were identified, 137 of which were duplicates and removed, leaving 3900 records that were screened for relevance based on title and abstract. Full texts were sought for twenty-two records that were deemed relevant to this review topic; full texts were obtained for 19 of these, and the remaining three were available as abstracts only, which were assessed for eligibility but were not included in this review due to breaches of the inclusion criteria. An additional two full texts were identified from screening the references of eligible studies. In total, seven studies, reported in ten records, were included. The ten records comprised eight journal articles (two of these reported the same study), one clinical trial record and one PhD thesis, each of which reported details of one of the seven included studies (Figure 1).

A table of excluded records and exclusion reasons is shown in Supplementary File S3.

### 3.1. Study Characteristics

Table 1 summarises study characteristics. The seven included studies were published between 2012 and 2020. Three studies were conducted in the USA, and one each in the UK, Sweden, New Zealand and Hong Kong. The studies included one non-randomised controlled trial, one interrupted time series and five before–after studies. The sample sizes ranged from four to 56. Participants’ mean age in each study was ≤19 years. Participant diagnoses included, for example, autism, Down syndrome, and attention deficit hyperactivity disorder (ADHD). Some studies described participants’ diagnoses more broadly, for example, intellectual or developmental disabilities and behavioural conditions. The diagnoses terminologies used in each study have been retained. In five studies, participants attended a specialist school; one study included participants from specialist education classrooms at a middle school in the USA, and in another study, participants attended an after-school club at a school that provides therapeutic specialist education for children with autism. Outcomes included measures of total food intake, nutrient intake, FV intake (selection and consumption), water intake and chocolate/confectionery consumption.

### 3.2. Intervention Descriptions

All interventions included were multi-component. The interventions were mapped to elements from the TIDieR-PHP [24] and AACTT [23] frameworks in Supplementary File S4 (data extraction form), which also contains all extracted results. The types of intervention are grouped into four categories: (1) modifications to the dining environment, (2) sensory exploration, (3) health promotion and (4) social reinforcement, as discussed below.

#### 3.2.1. Modifications to the Dining Environment

Two studies focused on dining modifications during the lunch period. Both studies were conducted with adolescents and young adults.

In one study (“Healthy plate” intervention) [26], regular dining plates were replaced with healthy eating plates showing how much of each food group should be eaten for a balanced meal. The ‘Healthy plate’ contained coloured pictures of three key food groups: green for vegetables, brown for potatoes, rice, bread/pasta and red for meat, fish or beans. The purpose of the healthy eating plate and the role of different nutrients were explained to all students at the start of each new school year by the health education teacher. During lunch, staff were encouraged to discuss the purpose of the plate and comment on missing foods. The healthy eating plate acted as a daily reminder of what food and the amount that should be on the plate. This was the only study that included a separate control group from two other schools. Digital photographs of each plate were taken and used to calculate the number of food items and food categories on the plates and the percentage of servings (normal-sized, overfilled, or half or less of a normal serving) amongst participants from the intervention (n = 27) and control groups (n = 62). The serving bowls were continuously refilled to make them as similar as possible, and the different foods were presented in identical bowls. To ensure fidelity of the comparison between intervention and control group participants, neither group was provided with healthy eating plates during the comparison (healthy eating plates had previously been available to the intervention group during lunch periods, but these were unavailable during the comparison/evaluation; hence, they were expected to have a lasting effect on food selection). No significant differences were found in relation to vegetable intake between the intervention and control groups (mean intake was 163 vs. 201 g, respectively, *p* = 0.208).

The second study (“Smarter Lunchroom” intervention) [27], which employed an interrupted time series design, involved a larger sample (n = 51 enrolled; n = 43 analysed) and used a behavioural economics approach involving multiple modifications in the dining room, including re-positioning of food items (i.e., fruit to the start of the food service line, peanut butter and jelly sandwiches to the back, and desserts behind the counter rather than at eye level), reduction of dessert portion sizes and swapping white bread for wholemeal. Improved food choices were expected to occur through these changes. Prior to the intervention, the menu was communicated to students through words and picture communication symbols. Peanut butter and jelly sandwiches continued to be available daily for students who had a very restricted food selection. Teachers were trained to support students in making autonomous food choices and adjust to changes in the dining hall layout, and teachers ate lunch with students to provide the support and supervision needed. Fidelity to the layout changes was monitored on separate occasions throughout the intervention period. Additionally, students who worked in the food serving area were supported to adapt to the dining room changes, and the ability of students and staff to serve the food as per the intervention protocol was monitored. Like the previous study, data collection methods were via digital photography to measure food selection and plate waste. These assessments took place for five consecutive days during the same week of the menu cycle for direct comparisons. Significant effects favouring the intervention were reported for canned fruit selection (relative risk (RR) = 2.37, 95% confidence interval (CI) 1.11, 5.08, *p* = 0.03) and canned fruit consumption (RR = 2.55, 95% CI 1.18, 5.54, *p* = 0.02). The mean percentage of FV servings wasted from those selected decreased by 9.4%, *p* = 0.04, and 9.0%, *p* = 0.03, respectively. Selection and consumption of whole grains significantly increased post-intervention (mean increase in the number of servings = 0.44, 95% Cl 0.14, 0.73; 0.38, 95% Cl 0.11, 0.65, respectively). There was a significant decrease in selection and consumption of refined grains (mean decrease in the number of servings = −0.33, 95% Cl −0.56, −0.11; −0.31, 95% Cl −0.51, −0.10, respectively). There were significant reductions in the selection and consumption of raw vegetable side dishes (RR = 0.54, 95% CI 0.41, 0.70, *p* = 0.001; RR = 0.68, 95% CI 0.49, 0.95, *p* = 0.02, respectively).

#### 3.2.2. Sensory Exploration

The intervention in two studies involved sensory exploration, where participants had the opportunity to interact with food that had different sensory qualities during snack times. This was intended to desensitise the pupils, who were all autistic, to new foods. Both studies employed a before–after design with each participant acting as their own control. One of these studies (“Sensory Snack Time” intervention) [9] delivered a whole-class sensory-based feeding intervention to primary school children (n = 23 enrolled, n = 19 analysed). Four to eight foods were presented during each session in hierarchies according to the sensory qualities of each food item. Across the 12-week intervention period, a total of 52 foods in 17 categories of food group and texture were presented. Clear plastic bags were used to present the food to avoid distractions from food branding. The intervention was delivered by school staff (teachers and special needs professionals). Prior to intervention delivery, the school dietitian and a language therapist led a one-hour staff training session based on a sensory approach to restrictive eating. The dietician attended at least 30% of the intervention sessions to ensure fidelity. Staff recorded the number of foods that each child selected and ate without prompting before and after the intervention. Teachers also completed the Brief Autism Mealtime Behaviour Inventory (BAMBI) questionnaire [31] pre- and post-intervention. Following the intervention, the mean number of foods tried by participants was significantly higher in seven food categories (*p* ≤ 0.05), and the number of participants who ate at least one item in a food category also increased for eight of the categories (*p* ≤ 0.05), compared to baseline. Teacher-reported BAMBI outcomes indicated lower Total, Food Selectivity, and Food Refusal scores, as well as lower Disruptive Mealtime Behaviour score and Severity score (all *p* ≤ 0.05); lower scores indicate less problematic mealtime behaviours. All the extracted results are available in Supplementary File S4 (data extraction form).

In the second study (“Physical Food Transformation” intervention) [29] participants were children and adolescents with autism (n = 56 enrolled). In this study, FVs were physically transformed to encourage enhanced acceptance. Examples of food transformations included turning bananas into ice cream, courgettes and sweet potatoes into chips, apples and kiwis into popsicles, and carrots into juice. In the first and fourth sessions, foods were prepared according to their original appearance, apart from being cut into chunks to make them easier to eat. In the second and third sessions, the food samples were transformed so that their appearance, and in some cases their taste and texture, were different. Details of any training, intervention delivery or fidelity were not reported. The outcome measures were FV acceptance and habitual FV consumption. FV acceptance was defined as a child putting food in their mouth and swallowing it. Habitual FV consumption was assessed using data from a pre- and post-intervention questionnaire collected from parents/guardians in the first and final (fourth) week. Findings showed that although the children’s percentage acceptance increased for all FV transformations, the mean change was only statistically significant for bananas (mean percentage of consumption increased from 55.3 to 78.0 g from baseline to post-intervention, *p* < 0.05) and not for apples, kiwis, courgettes, carrots, or sweet potatoes. In relation to habitual FV consumption, parents/guardians reported an increase in banana, apple, kiwi, courgette and sweet potato consumption (*p* < 0.05).

#### 3.2.3. Health Promotion

The interventions in two before–after studies involved more general health promotion programmes, focusing on a combination of physical activity and dietary habits.

One of the studies (the “Mind, Exercise, Nutrition…Do It! (MEND)” programme) [28] involved children and adolescents (n = 22 enrolled, n = 17 analysed). The intervention was a modified version of a family-based weight programme that was found to be effective in the general population of CYP. The 10-week programme comprised 18 sessions focusing on physical activity, healthy eating and motivational skills. A community paediatric physiotherapist and dietician delivered the sessions with support from teachers, teacher aids, senior management and social workers. Additional support was provided from health promotion and public health nurses. The sessions were held twice a week, and for the nutrition aspect, parents/carers attended a one-hour nutrition session. Children were taught about increasing water intake, MEND-friendly foods and the importance of eating breakfast. Processes to enable families to remain engaged in the programme included identifying times that suited families, starting and finishing sessions on time, availability of transport and briefing sessions for parents/carers, teachers, coordinators and project staff. Quantitative and qualitative data were collected. Quantitative data were collected from a 14-item nutrition questionnaire collecting information on frequency of consumption, for example, breakfast, fizzy drinks, confectionery and cooking fresh food. This was completed by parents and carers before and after the programme and 24 weeks post-programme. Data was only reported on confectionery and chocolate consumption. From pre- to post-intervention, the number of parents/carers who reported that their child consumed confectionery and chocolate a few times a week dropped from 44% pre-intervention to 33% post-intervention, and the number of parents/carers who reported their child’s consumption had reduced to rarely increased from 31% pre-intervention to 50% post-intervention. Twenty-four weeks post-intervention, over 80% of the parents/carers reported that their child consumed confectionery and chocolates once a week or rarely.

In the second study (“The Healthy Living Mentoring Program”) [20], adolescent participants (n = 25 enrolled, n = 14 analysed) from segregated special education classrooms at a local middle school were each assigned a mentor who provided structured, interactive sessions to promote sustainable healthy lifestyle choices. Mentors (n = 39) were college students from a local university. Before the intervention, mentors attended a 2.5 h training session led by an adapted physical education specialist, who provided an overview of the programme, their responsibilities, outcome measures, communication strategies, and session self-report requirements. Sessions were weekly and lasted 60–70 min. Classroom teachers worked with the mentors to increase daily physical activity and monitor mentees’ healthy eating behaviours. To ensure fidelity, the programme coordinator supervised all mentoring sessions, and mentors completed online session monitoring forms. This intervention took place during the lunch hour. Nutritional outcomes were daily water and FV intake assessed by participant self-report. Following the intervention, mean participant water intake (number of 8 oz cups/day) increased by 4.36 cups (95% CI 0, 8, *p* < 0.0001); similarly, there was an increase in the percentage of pupils reporting they ate FVs daily (7% and 86%, respectively; *p* < 0.0001).

#### 3.2.4. Social Reinforcement

This study aimed to improve FV intake through a team game intervention (“The Good Nutrition Game”) [30]. The intervention was delivered at an after-school programme for autistic CYP and focused on the snack time. Participants were adolescents (n = 10 enrolled, n = 4 analysed). For the intervention, participants were separated into teams. Teams were given easy-to-eat FV chunks and invited to play a game where one point was won for each bite of fruit or vegetable taken. Scores were recorded on a scoreboard that was displayed at the front of each room. Each session lasted 15 min. The winning team won a small prize. The intervention was delivered by a researcher with support from classroom teachers and assistants. Intervention fidelity was ensured by a review of the protocol and procedural checklist by the clinical supervisor, teachers, and staff. Before the session, the researcher prepared all materials (e.g., snacks, game resources) according to the set guidelines. The outcome measure was the number of bites (self-reported by the students) of FVs at baseline (during normal snack time) and during the intervention. Consumption, defined as the number of bites of FVs, increased from a mean of 6.2 to 13.8 pre- to post-intervention (statistical significance was not reported).

## 4. Discussion

### 4.1. Summary of Findings

A key finding was the small number of studies reporting on interventions to support healthier food choices in CYP attending specialist schools. Only seven studies met the inclusion criteria. Most studies included adolescents; only one study focused on primary school-aged children. In two of the studies, the interventions were delivered during the lunch period; three studies focused on the break/snack time in school settings, and two studies focused on more general sessions that were not lunch or break/snack specific. Each study reported the potential mechanisms by which the intervention was considered to promote healthy eating and included a reminder of what proportions of different food groups should be on a pupil’s lunch plate; ‘nudging’ pupils to make healthier food choices; addressing sensory food preferences; increasing self-efficacy to make healthier food choices; and using competition and peer support to motivate students to consume more FVs. The most commonly reported nutritional outcome across studies was fruit and vegetable (FV) consumption. Although several interventions showed some evidence of increased FV intake, the findings were inconsistent, reflecting substantial variation in both intervention approaches and methods of assessing dietary intake—including self-report, parental questionnaires, and observational measures. For example, the Healthy Plates [26] intervention did not detect statistically significant differences in vegetable intake between intervention and control groups, whereas the Smarter Lunchrooms [27] approach reported significant increases in canned fruit selection and consumption but also noted a reduction in raw vegetable intake. Sensory-based interventions produced mixed results: Sensory Snack Time [9] showed a significant increase in the number of foods tried, whereas the Physical Food Transformation [29] intervention reported effects only for bananas. The Healthy Living Mentoring Programme [20] found a statistically significant increase in the proportion of pupils consuming FVs, while the Good Nutrition Game [30] reported increased bites of FVs, though only descriptive results were provided. These studies employed different intervention models, outcome measures (bites, proportion, etc.), and dietary assessment tools, making it difficult to draw conclusions. Furthermore, the interventions were implemented at different times across the school day, such as breaktime and lunchtime, making comparisons difficult. This variability highlights the need for more consistency in methodological approach and outcome measure selection to allow meaningful comparison of intervention effects.

### 4.2. Strengths and Limitations

To our knowledge, this is the first review to identify interventions to support healthier food choices for CYP attending specialist schools. A strength is that a proportion of records were double screened, and all extracted data were checked for accuracy and completeness, ensuring robustness of the methods. Published peer-reviewed and grey literature were assessed with no restrictions to time periods or geographical location. A narrative synthesis allowed a detailed description of the included studies, which had a range of study designs, participant ages, conditions, and school-level interventions. As this was a scoping review, the quality of included studies was not assessed using standardised tools. The aim was to describe the literature, identify key concepts and identify evidence gaps, rather than evaluate intervention effectiveness.

The included studies had a number of limitations. The study samples were small, potentially limiting the ability to detect statistically significant effects [32] and limiting the representativeness of findings. Most studies did not have an independent control group or random allocation to the intervention or control group, making it difficult to draw a conclusion about any correlation between the intervention and the selected outcome. In several studies, a large number of outcome measures were analysed, increasing the probability of detecting significant differences by chance [33]. All included studies reported short-term outcomes only, mainly using self-reported measures. Information about sustained intervention outcomes and impacts was unavailable. Selected outcome measures tended to focus on nutritional rather than wider health outcomes, meaning that longer-term impacts of intervention on health and wellbeing could not be assessed. There was a lack of qualitative evidence, which may provide insight into intervention acceptability and feasibility and the mechanisms by which healthy eating interventions are effective. Only one study included qualitative and quantitative methods [28]. There was no mention in any study about the involvement and engagement of stakeholders in the intervention development and study design. Several of the studies used interventions that may be more appropriate for use with more cognitively able CYP compared to those with moderate/severe learning disabilities (for example, the use of a healthy plate) or more complex needs, including physical disabilities or physical eating/drinking difficulties. Most interventions included multiple components, yet it was unclear which elements were associated with positive effects. Non-English publications were excluded; this may have introduced language bias and limited the diversity of perspectives, interventions and contexts considered in this review.

### 4.3. Future Research and Conclusion

The included studies identify some intervention components and outcome measures which may be relevant for future multi-component intervention development work. However, due to the small sample sizes and heterogeneity of the study designs and outcomes, definitive conclusions cannot be drawn. Further rigorous research to understand more about factors influencing healthy food choices amongst specialist school pupils and about opportunities for interventions to improve healthy food choices is required ahead of intervention feasibility, acceptability, and efficacy work. Intervention development relating to food and drink provision across the school day and as part of a whole-school food systems approach is needed to achieve maximum impact on CYP’s food and drink choices. Both primary and secondary specialist school settings must be included to ensure findings are relevant to all specialist school pupils. Future school food intervention research needs to involve multiple stakeholders throughout the lifecycle of the research to determine research priorities and ensure the relevance of the research to those for whom the research is being conducted. This process should involve school staff, parents and pupils, including the co-development of study aims, agreement on acceptable methods, collaborative intervention design, identifying meaningful outcomes and an iterative review of both the process and interpretation of findings. This finding of the need to include CYP attending specialist schools in research aligns with a commentary by Schenkelberg et al., 2023, who identified that CYP with learning difficulties are under-represented in health behaviour research [34]. They specifically emphasise the need for young children to be included in obesity-prevention interventions [34]. Intervention co-production will help the development of effective specialist school food interventions that are acceptable, feasible, and sustainable in the context of resource limitations.

Due to the lack of research in this area, identifying practice and policy recommendations is challenging. However, prioritising co-production and ensuring this population is given greater prominence is essential for improving school food provision and ultimately dietary intakes.

This review highlights a crucial need for the development of new multi-component interventions co-produced with stakeholders to improve the dietary intakes of CYP attending specialist schools. To improve the dietary intakes for CYP attending specialist schools, school food interventions must accommodate CYP’s diverse neurodevelopmental, physical, and health needs.

## Figures and Tables

**Table 1:** Characteristics of included studies and their participants.

Author Surname, Year	Country	Study Design	Intervention Group	Intervention Name and Time	Control Group	Intervention Comparator	Relevant Outcomes
Flygare-Wallen, 2013 [26]	Sweden	Non-randomised controlled trial	**n** = 27 (analysed) **Age (years)** = 16–21 **Diagnoses:** mild/moderate intellectual disabilities, Down syndrome **Setting:** upper secondary school	Multifactorial intervention using the ‘Plate Model’ Lunchtime	**n** = 62 **Age (years)** = 16–21 **Diagnoses:** mild/moderate intellectual disabilities, Down syndrome **Setting:** a private secondary school for children with intellectual disabilities and a regular municipal school	Traditional canteen	Lunch eaten (g/Kcals); Carbohydrates eaten (g); Vegetables eaten (g); Plate waste (≥2.5 g) Macronutrients: (carbohydrates (%), fat (%), protein (%))
Hubbard, 2015 [27]	USA	Interrupted time series	**n** = 43 (analysed) **Age (years)** = 11–22 **Diagnoses:** intellectual and developmental disabilities, range of secondary emotional, mental health and behavioural conditions, including autism **Setting:** private specialist residential school	Smarter lunchroom intervention Lunchtime	NA	Pre–post-intervention	Selection and consumption of food (KJ and g); daily servings of fruit, vegetables, whole and refined grains
Galpin, 2018 [9]	England, UK	Before–after study	**n** = 19 (analysed) **Age (years)** = 4–10 **Diagnoses:** autism with communication difficulties and/or behavioural challenges and complex needs, including additional learning difficulties **Setting:** Primary school	Sensory snack time: a whole class sensory feeding intervention Breaktime	NA	Pre–post-intervention	Number of foods tried, number of participants who ate at least one item in a food category (fruit, vegetables, protein, and carbohydrate-containing food with different sensory qualities)
Hinckson, 2012 [28]	New Zealand	Before–after study	**n** = 17 (analysed) **Age (years)** = 8–18 **Diagnoses:** autism, global developmental delay, autistic–obsessive behaviours and hyperactivity, Down syndrome, intellectual disability **Setting:** specialist schools (n = 2)	Modified MEND (‘‘Mind, Exercise, Nutrition. Do It!’’) programme School 1: two daytime sessions on Wednesday and Thursday, 1100–1300 School 2: two evening sessions on Monday and Thursday, 1730–1930	NA	Pre–post-intervention	Frequency of chocolate and confectionery consumption Parent/carer reported nutritional behaviours
Chung, 2020 [29]	Hong Kong	Before–after study	**n** = 56 (enrolled; N analysed not reported) **Age (years)** = 8–15 **Diagnosis:** autism **Setting:** specialist school (n = 3)	Physical Food Transformation Breaktime	NA	Pre–post-intervention	Percentage of consumption of banana, apple, kiwi fruit, courgette, carrot, sweet potato Parent/guardian reported child fruit and vegetable consumption in the prior three months (assessed on a 5-point Likert scale: 1, ‘never/rarely’; 5, ‘almost every meal’)
Cassey, 2016 [30]	USA	Before–after study	**n** = 4 (analysed) **Age (years)** = 14–19 **Diagnoses:** autism, ADHD, obsessive–compulsive disorder pervasive developmental disorder, anxiety disorder, oppositional defiant disorder **Setting:** school providing therapeutic special education	The ‘Good Nutrition Game’ After-school club	NA	Pre–post-intervention	Number of bites of fruits and vegetables
Jihoun, 2019 [20]	USA	Before–after study	**n** = 14 (analysed) **Age (years)** = 12–15 **Diagnoses:** autism, intellectual disability, obsessive–compulsive disorder, ADHD, pervasive developmental disorder, anxiety disorder, oppositional defiant disorder **Setting:** segregated special education class in a middle school	‘The Healthy Living Mentoring Program’ Lunchtime	NA	Pre–post-intervention	Self-reported daily water, fruit and vegetable intake

Abbreviations: NA = not applicable.

## Data Availability

Supporting data can be found in the supplementary files.

## Supplementary Materials

The following supporting information can be downloaded at: https://www.mdpi.com/article/10.3390/nu18071037/s1, Supplementary File S1: PRISMA-ScR checklist; Supplementary File S2: Medline search strategy; Supplementary File S3: Table of excluded studies; Supplementary File S4: data extraction form.

## Abbreviations

The following abbreviations are used in this manuscript:

AACTT	Action, Actor, Context, Target, Time
ADHD	Attention deficit hyperactivity disorder
CYP	Children and young people
FVs	Fruit and vegetables
g	Grams
KJ	Kilojoules
NA	Not applicable
PCC	Population, Concept and Context
PRISMA-P	The Preferred Reporting Items for Systematic Reviews and Meta-analyses Protocols
PRISMA-ScR	PRISMA Scoping Review
SENs	Special educational needs
TIDieR-PHP	Template for Intervention Description and Replication for Population Health and Policy Interventions
