# Supplementary material for: A Scoping Review to Identify Interventions That Support Healthier Food Choices for Pupils in Specialist Schools
Source: Nutrients. 2026 Mar 25;18(7):1037. doi: 10.3390/nu18071037 (PMC13074712; doi:10.3390/nu18071037)
Supplement: Supplementary file 1 [file nutrients-18-01037-s001.zip › Supplementary Files S1-S3.pdf]

Supplementary files

Supplementary file S1 PRISMA-ScR checklist

Preferred Reporting Items for Systematic reviews and Meta-Analyses extension for Scoping Reviews (PRISMA-ScR) Checklist

SECTION	ITEM	PRISMA-ScR CHECKLIST ITEM	REPORTED ON PAGE #
**TITLE**			
Title	1	Identify the report as a scoping review.	1
**ABSTRACT**			
Structured summary	2	Provide a structured summary that includes (as applicable): background, objectives, eligibility criteria, sources of evidence, charting methods, results, and conclusions that relate to the review questions and objectives.	1
**INTRODUCTION**			
Rationale	3	Describe the rationale for the review in the context of what is already known. Explain why the review questions/objectives lend themselves to a scoping review approach.	2
Objectives	4	Provide an explicit statement of the questions and objectives being addressed with reference to their key elements (e.g., population or participants, concepts, and context) or other relevant key elements used to conceptualize the review questions and/or objectives.	2-3
**METHODS**			
Protocol and registration	5	Indicate whether a review protocol exists; state if and where it can be accessed (e.g., a Web address); and if available, provide registration information, including the registration number.	2
Eligibility criteria	6	Specify characteristics of the sources of evidence used as eligibility criteria (e.g., years considered, language, and publication status), and provide a rationale.	3
Information sources*	7	Describe all information sources in the search (e.g., databases with dates of coverage and contact with authors to identify additional sources), as well as the date the most recent search was executed.	3; Supplementary file S2
Search	8	Present the full electronic search strategy for at least 1 database, including any limits used, such that it could be repeated.	Supplementary file S2
Selection of sources of evidence†	9	State the process for selecting sources of evidence (i.e., screening and eligibility) included in the scoping review.	3
Data charting process‡	10	Describe the methods of charting data from the included sources of evidence (e.g., calibrated forms or forms that have been tested by the team before their use, and whether data charting was done independently or in duplicate) and any processes for obtaining and confirming data from investigators.	3
Data items	11	List and define all variables for which data were sought and any assumptions and simplifications made.	3; Supplementary file 4
Critical appraisal of individual sources of evidence§	12	If done, provide a rationale for conducting a critical appraisal of included sources of evidence; describe the methods used and how this information was used in any data synthesis (if appropriate).	NA

Synthesis of results	13	Describe the methods of handling and summarizing the data that were charted.	3
**SECTION**	**ITEM**	**PRISMA-ScR CHECKLIST ITEM**	**REPORTED ON PAGE #**
**RESULTS**			
Selection of sources of evidence	14	Give numbers of sources of evidence screened, assessed for eligibility, and included in the review, with reasons for exclusions at each stage, ideally using a flow diagram.	Figure 1, page 4
Characteristics of sources of evidence	15	For each source of evidence, present characteristics for which data were charted and provide the citations.	Table 1, pages 6-9
Critical appraisal within sources of evidence	16	If done, present data on critical appraisal of included sources of evidence (see item 12).	NA
Results of individual sources of evidence	17	For each included source of evidence, present the relevant data that were charted that relate to the review questions and objectives.	10-13
Synthesis of results	18	Summarize and/or present the charting results as they relate to the review questions and objectives.	Table 1, pages 6-9; pages 10-13
**DISCUSSION**			
Summary of evidence	19	Summarize the main results (including an overview of concepts, themes, and types of evidence available), link to the review questions and objectives, and consider the relevance to key groups.	Pages 10-13
Limitations	20	Discuss the limitations of the scoping review process.	14
Conclusions	21	Provide a general interpretation of the results with respect to the review questions and objectives, as well as potential implications and/or next steps.	14-15
**FUNDING**			
Funding	22	Describe sources of funding for the included sources of evidence, as well as sources of funding for the scoping review. Describe the role of the funders of the scoping review.	15

JBI = Joanna Briggs Institute; PRISMA-ScR = Preferred Reporting Items for Systematic reviews and Meta-Analyses extension for Scoping Reviews.

* Where *sources of evidence* (see second footnote) are compiled from, such as bibliographic databases, social media platforms, and Web sites.

† A more inclusive/heterogeneous term used to account for the different types of evidence or data sources (e.g., quantitative and/or qualitative research, expert opinion, and policy documents) that may be eligible in a scoping review as opposed to only studies. This is not to be confused with *information sources* (see first footnote).

‡ The frameworks by Arksey and O'Malley (6) and Levac and colleagues (7) and the JBI guidance (4, 5) refer to the process of data extraction in a scoping review as data charting.

§ The process of systematically examining research evidence to assess its validity, results, and relevance before using it to inform a decision. This term is used for items 12 and 19 instead of "risk of bias" (which is more applicable to systematic reviews of interventions) to include and acknowledge the various sources of evidence that may be used in a scoping review (e.g., quantitative and/or qualitative research, expert opinion, and policy document).

From: Tricco AC, Lillie E, Zarin W, O'Brien KK, Colquhoun H, Levac D, et al. PRISMA Extension for Scoping Reviews (PRISMA-ScR): Checklist and Explanation. Ann Intern Med. ;169:467–473. doi: 10.7326/M18-0850

Supplementary file S2 Medline search strategy

Search number	Search term	Number of results
1	Autism Spectrum Disorder/ or Attention Deficit Disorder with Hyperactivity/	57868
2	Child Development Disorders, Pervasive/ or Autistic Disorder/ or Asperger Syndrome/	33865
3	Cerebral Palsy/	24724
4	Cognition Disorders/ or Cognitive Dysfunction/	108604
5	Deglutition Disorders/	25102
6	Developmental Disabilities/	23040
7	Disabled Persons/	49523
8	Down Syndrome/	27078
9	Dyslexia/	8657
10	Hearing Disorders/	14701
11	Hearing Loss/	21327
12	Intellectual Disability/	61503
13	Learning Disabilities/	14794
14	motor disorders/ or motor skills disorders/	4455
15	Neurodevelopmental Disorders/	5435
16	Vision Disorders/	31490
17	(disab* or autis* or developmental disorder* or neurodevelopmental disorder* or (special adj3 (educ* or need*))).ti. or (special adj3 (educ* or need*)).ab. /freq=3 or autis*.ab. /freq=3 or developmental disorder*.ab. /freq=3 or neurodevelopmental disorder*.ab. /freq=3	124951
18	or/1-17	509408
19	Schools/	54804
20	Education, Special/	9177
21	School Health Services/	18688
22	school teachers/	2982
23	(counseling/ or directive counseling/) and educat*.ti,ab,kf.	6887

24	school*.mp.	403429
25	or/19-24	415611
26	Health Behavior/ or Behavior Therapy/ or Health Promotion/	162886
27	Choice Behavior/ or Food Preferences/	50141
28	Health Education/	64593
29	Nutrition Policy/	11004
30	Dietary Services/	1420
31	Food Services/	6321
32	nutrition therapy/ or diet therapy/ or caloric restriction/ or diet, reducing/	32710
33	Child Nutrition Disorders/dh, pc or exp Obesity/dh, pc or Metabolic Diseases/dh, pc or Overweight/dh, pc	33865
34	exp Cardiovascular Diseases/pc and exp Cardiovascular Diseases/dh	1512
35	(intervention* or program* or promot*).ti.	723375
36	(educ* adj4 (program* or intervention*)).ti,ab,kf.	105235
37	((choice* or habit*) adj4 (healthy or diet* or food)).ti,ab,kf.	39997
38	(food* adj3 select*).ti,ab,kf.	6163
39	(eating* adj3 pattern*).ti,ab,kf.	4730
40	or/26-39	1095339
41	(food* or nutri* or diet or eat*).ti.	441634
42	(food* or nutri* or diet* or eat*).ab. and ((healthy or behav*).ti. or (healthy and lifestyle).ab.)	62101
43	food*.ab. /freq=2 or nutri*.ab. /freq=2 or diet*.ab. /freq=2 or eat*.ab. /freq=2	688011
44	exp food/	1539475
45	Taste/	27045
46	feeding behavior/ or food fussiness/ or food preferences/	108070
47	Diet, Healthy/ or Diet/	201763
48	Nutritive Value/	16707

49	nutritional requirements/ or recommended dietary allowances/	22311
50	or/41-49	2221448
51	18 and 25 and 40 and 50	128
52	"Education of Intellectually Disabled"/ and (adolescent/ or child/ or (child* or adolescen*).mp.) and 40 and 50	28
53	(student* or pupil* or teacher* or special education*).ti,ab,kf. and (adolescent/ or child/ or (child* or adolescen*).mp.) and 18 and 40 and 50	48
54	51 or 52 or 53	176

Supplementary file S3: Table of excluded studies

Record number	Reference	First recorded exclusion reason
1	Hubbard KL, Bandini LG, Folta SC, Wansink B, Must A. The Adaptation of a School-based Health Promotion Programme for Youth with Intellectual and Developmental Disabilities: A Community-Engaged Research Process. Journal of Applied Research in Intellectual Disabilities. 2014 Nov;27(6):576-90.	Wrong outcome
2	Breda C, Santero S, Conti MV, Cena H. Programs to manage Food Selectivity in individuals with Autism Spectrum Disorder. Nutrition Research Reviews. 2024 Feb 22:1-34.	Wrong outcome
3	Hubbard KL, Bandini LG, Folta SC, Wansink B, Must A. The Adaptation of a School-based Health Promotion Programme for Youth with Intellectual and Developmental Disabilities: A Community-Engaged Research Process. Journal of Applied Research in Intellectual Disabilities. 2014 Nov;27(6):576-90.	Wrong outcome
4	Banire B, Khowaja K, Mansoor B, Qaraqe M, Al Thani D. Reality-based technologies for children with autism spectrum disorder: A recommendation for food intake intervention. Personalized Food Intervention and Therapy for Autism Spectrum Disorder Management. 2020:679-93.	Wrong study design
5	מרגליות ט, הופמן שמ. " Eating Onn"-A Joint Lunch Therapy Intervention/אכיל און-טיפול קבוצתי במסגרת ארוחת צהריים משותפת. כתב עת ישראלי לריפוי בעיסוק. 2015 Nov 1:H205-16.	Wrong outcome
6	Gast AH, Shepley C, Lane JD. Challenges and Successes in Addressing Food Selectivity in Children With Developmental Disabilities During Preschool Mealtimes. Infants & Young Children. 2020 Oct 1;33(4):300-12.	Wrong population
7	Flygare Wallen E., Mullerdorf M. & Marcus C. (2010). A school-intervention with increased physical activity and healthy food choices puts a halt to obesity in youth with intellectual disability. Obesity Reviews, 11(SUPPL. 1), 90.	Wrong outcome
8	Congdon M. Increasing food acceptance in the school setting for children with autism spectrum disorder using high probability requests sequences (Doctoral dissertation).	Wrong outcome
9	Buro AW, Gray HL, Kirby RS, Marshall J, Van Arsdale W. The BALANCE nutrition education intervention for adolescents with ASD: A formative study in a school setting. Research in Autism Spectrum Disorders. 2022 Mar 1;91:101912.	Wrong outcome
10	Buro AW. Feasibility of a Virtual Group Nutrition Intervention for Adolescents with Autism Spectrum Disorder (Doctoral dissertation, University of South Florida).	Wrong outcome
11	Bonito J, Boné M. Healthy food practical in children with intellectual disabilities. Atención primaria. 2014;46:26.	Wrong outcome

12	Bailey T, Thabtah F, Wright M, Tran DA. FoodKnight: a mobile educational game and analyses for obesity awareness of children. International Journal of Medical Engineering and Informatics. 2024;16(2):139-49.	Wrong population
13	Bailey, R. L., & Angell, M. E. (2005). Improving Feeding Skills and Mealtime Behaviors in Children and Youth with Disabilities. Education and Training in Developmental Disabilities, 40(1), 80–96.	Wrong concept
14	Clinicaltrials.gov. Healthy Eating ASD. 2022. Available from: https://clinicaltrials.gov/study/NCT04075552?term=NCT04075552&rank=1 [Accessed 09 01 2025].	Study withdrawn
